# Supplementary material for: Comparison of Antifungal Activity of Bacillus Strains against Fusarium graminearum In Vitro and In Planta
Source: Plants (Basel). 2022 Jul 31;11(15):1999. doi: 10.3390/plants11151999 (PMC9370729; doi:10.3390/plants11151999)
Supplement: Supplementary file 1 [file plants-11-01999-s001.zip › plants-1840967-Supplemental Figure .pdf]

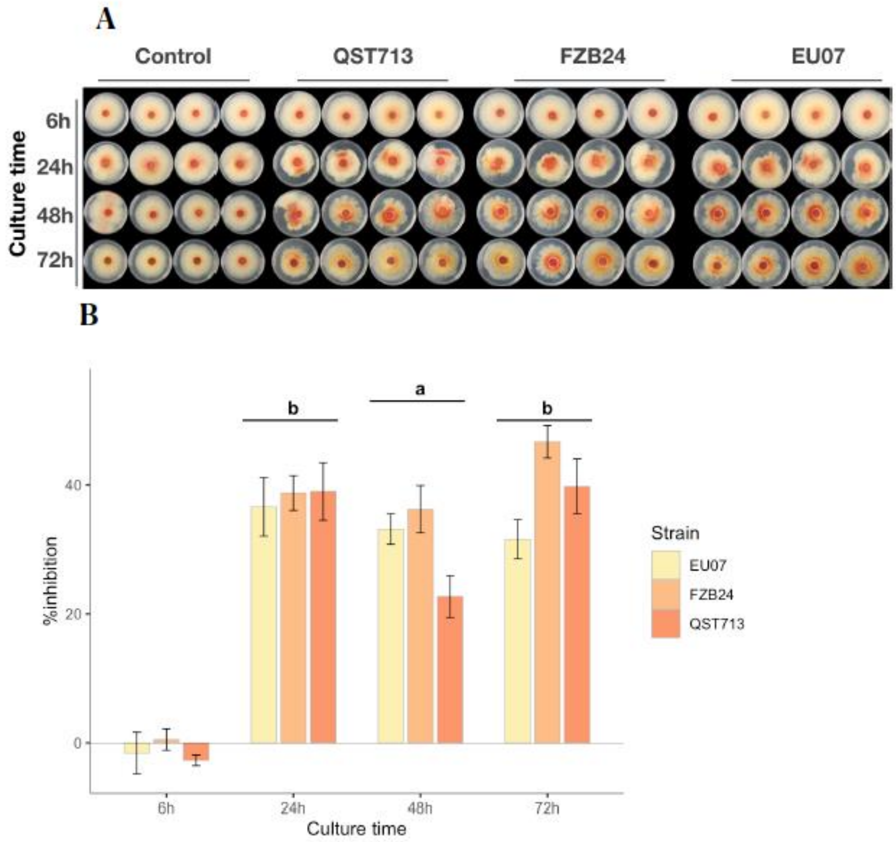

Supplemental Figure S1. Cell-free bacterial cultures inhibit fungal growth.

Fg-K1-4 growing on PDA/NA medium containing filtered bacterial broths. The fungus was grown on PDA/NA medium in the presence of the 100µl filtered bacterial broths obtained from QST713, FZB24 and EU07 after 6, 24, 48 and 72h of culture. B) The percentage inhibition of the fungal growth by the filtered broth of *Bacillus* strains obtained at different times (6h, 24h, 48h and 72h). Data were from one independent experiment of four replicates and shown as the mean ± SE. Experiment was repeated at least three times with similar results. Bars clusters with different letters were significantly different according to Tukey's Test (α < 0.05) following two-way ANOVA. N=48.

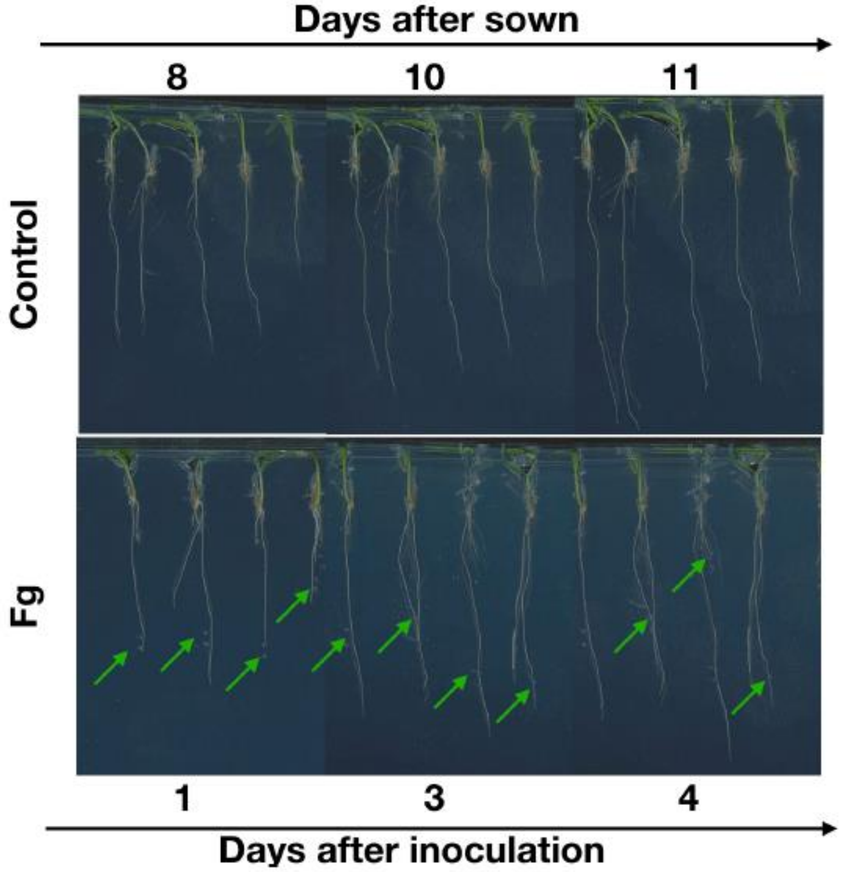

Supplemental Figure S2. Root infection assays with Bd-21.

Bd-21 roots displayed secondary growth from the crown after inoculation with *Fg*-K1-4. Data were from one independent experiment of 6-7 replicates. Experiments were repeated twice, and similar results were obtained. n=60. B) Trypan blue staining of the detached leaves of Bd-21. Adaxial view of leaves of Bd-21 non-infected (Control), infected with *Fg*-K1-4 (*Fg*) and infected with *Fg*-K1-4 and treated with *Bs* (Fg/Bs). Trypan blue staining was performed 4dpi. LC: Long cells, SC: Short Cells, P: Papilla, H: Hair, Hy: Hypha, Mc: Macroconidia, Nc: Necrotic damage. Scale bars = 100 µm.
